# Nudix hydrolases degrade protein-conjugated ADP-ribose

**DOI:** 10.1038/srep18271

**Published:** 2015-12-16

**Authors:** Casey M. Daniels, Puchong Thirawatananond, Shao-En Ong, Sandra B. Gabelli, Anthony K. L. Leung

**Affiliations:** 1 Department of Biochemistry and Molecular Biology, Bloomberg School of Public Health, Johns Hopkins University, Baltimore, MD 21205, USA; 2 Department of Biophysics and Biophysical Chemistry, Johns Hopkins University School of Medicine, Baltimore, MD 21205, USA; 3 Department of Pharmacology, University of Washington, Seattle, WA 98195, USA; 4 Department of Medicine, Johns Hopkins University School of Medicine, Baltimore, MD 21205, USA; 5 Department of Oncology, Johns Hopkins University School of Medicine, Baltimore, MD 21205, USA

## Abstract

ADP-ribosylation refers to the transfer of the ADP-ribose group from NAD^+^ to target proteins post-translationally, either attached singly as mono(ADP-ribose) (MAR) or in polymeric chains as poly(ADP-ribose) (PAR). Though ADP-ribosylation is therapeutically important, investigation of this protein modification has been limited by a lack of proteomic tools for site identification. Recent work has demonstrated the potential of a tag-based pipeline in which MAR/PAR is hydrolyzed down to phosphoribose, leaving a 212 Dalton tag at the modification site. While the pipeline has been proven effective by multiple groups, a barrier to application has become evident: the enzyme used to transform MAR/PAR into phosphoribose must be purified from the rattlesnake *Crotalus adamanteus* venom, which is contaminated with proteases detrimental for proteomic applications. Here, we outline the steps necessary to purify snake venom phosphodiesterase I (SVP) and describe two alternatives to SVP—the bacterial Nudix hydrolase *Ec*RppH and human *Hs*NudT16. Importantly, expression and purification schemes for these Nudix enzymes have already been proven, with high-quality yields easily attainable. We demonstrate their utility in identifying ADP-ribosylation sites on Poly(ADP-ribose) Polymerase 1 (PARP1) with mass spectrometry and discuss a structure-based rationale for this Nudix subclass in degrading protein-conjugated ADP-ribose, including both MAR and PAR.

ADP-ribosylation is a post-translational modification (PTM) implicated in a number of disease states, including cancer, diabetes, and a range of neuropathologies^1^. This protein modification is synthesized by ADP-ribosyl transferases, commonly known as poly(ADP-ribose) polymerases (PARPs), which transfer the ADP-ribose (ADPr) group from NAD^+^ to protein acceptor amino acids in monomeric (mono(ADPr), MAR) and/or polymeric (poly(ADPr), PAR) form^2^,^3^. Identification of specific amino acid acceptors of ADPr group(s), and therefore characterization of the cellular role played by this important protein modification, has been hampered by the lack of a robust, universal method for identifying ADP-ribosylation sites in the proteome. This need has lately been addressed by three different methods^4^,^5^,^6^,^7^,^8^,^9^,^10^,^11^,^12^,^13^,^14^, all of which involve the removal of any ADPr subunits beyond the protein-proximal monomer, followed by identification of the ‘tag’ left behind at the ADPr conjugation site (reviewed in ref. 14). One of these methods, hydrolysis of MAR/PAR down to its phosphoribose attachment site, relies upon the pyrophosphatase activity of snake venom phosphodiesterase I (SVP) from *Crotalus adamanateus*, which can be purchased in a partially purified form that requires further purification for use against ADP-ribosylated proteins^3^,^5^,^6^. Unfortunately, this complicated purification scheme ultimately results in a high level of prep-to-prep variability, likely due to the inherently variable protein source (snake venom) as well as the number of purification steps involved. In an effort to identify a more reliable tool for the degradation of protein-conjugated MAR/PAR to phosphoribose, we describe here the characterization of candidate enzymes from the Nudix hydrolase superfamily.

The Nudix hydrolase superfamily catalyzes hydrolysis of **Nu**cleoside **Di**phosphates linked to other moieties (“**X**”). Most Nudix families contribute to cellular ‘housekeeping’ through the breakdown of a wide range of nucleoside diphosphate derivatives^15^. One of these diphosphate containing compounds is ADPr^16^,^17^,^18^,^19^, a molecule which is known to accumulate in cells with potentially cytotoxic effects by: (1) altering calcium entry into cells via channel gating, thus affecting membrane depolarization^20^, (2) serving as a neurotransmitter in primate and murine colons^21^, and (3) spontaneously modifying proteins^22^, potentially altering intracellular post-translational signaling. Without Nudix hydrolase activity, free ADPr would amass during the breakdown of PAR^23^,^24^, as a side product of tRNA synthesis^25^, following NAD^+^ glycohydrolysis^26^, following deacetylation of O-acetyl-ADPr^27^, or through the breakdown of the signaling molecule cyclic ADPr^28^. Accordingly, ADPr degrading Nudix enzymes are broadly conserved, with humans possessing at least six distinct ADPr pyrophosphatases (ADPrases) responsible for hydrolyzing ADPr to AMP and phosphoribose^18^,^29^. In light of this known ADPrase activity, we reasoned that Nudix hydrolases could potentially replace SVP for generating phosphoribose tags at ADP-ribosylation sites in the proteomics pipeline described above, and screened bacterial Nudix hydrolases for comparable hydrolysis activity of protein-conjugated ADPr. These efforts lead to the identification of the RNA 5′ pyrophosphohydrolase (RppH) from *Escherichia coli* (*Ec*RppH) as capable of degrading protein-conjugated ADPr, including both MAR and PAR. From a structural and biological perspective, this finding was unexpected as *Ec*RppH is an RNA decapping enzyme, and not an ADPrase^30^. While we were characterizing this novel activity of *Ec*RppH, a human Nudix hydrolase NudT16 (*Hs*NudT16) also known to decap RNA^31^, was shown to degrade protein-conjugated MAR and PAR^13^. In this study, we provide a structure-based rationale for the inability of Nudix ADPrases to degrade protein-conjugated ADPr, in contrast to Nudix RNA decapping enzymes, and demonstrate the use of both *Ec*RppH and *Hs*NudT16 in the identification of protein ADP-ribosylation sites by mass spectrometry.

## Materials and Methods

### Mutagenesis of PARP1 to the E988Q catalytically deficient mutant

pET28 6xHis-PARP1 was a gift from Dr. John Pascal and served as the template for mutagenesis into the mono(ADP-ribose) restricted mutant of PARP1, E988Q. Primers used for mutagenesis: Forward: GACACCTCTCTACTATATAAC**C**AGTACATTGTCTATGATATTGC, Reverse: GCAATATCATAGACAATGTACT**G**GTTATATAGTAGAGAGGTGTC.

### Expression and purification of wild-type (WT) and E988Q His-PARP1

WT and E988Q His-PARP1 was expressed and purified as previously described^5^.

### Purification of SVP

SVP was purified as previously described^5^ from starting material obtained from United States Biological, catalog number P4072, lot number L14030507 C14062702. Briefly, SVP powder from a vial was dissolved into 1 mL of loading buffer (10 mM Tris-HCl pH 7.5, 50 mM NaCl, 10% glycerol) and then loaded onto a pre-equilibrated 1 mL HiTrap blue sepharose column (GE, 17-0412-01), washed with 5 column volumes of loading buffer and then 5 column volumes of elution buffer (10 mM Tris-HCl pH 7.5, 50 mM NaCl, 10% glycerol, 150 mM potassium phosphate). Desired fractions were pooled, dialyzed against loading buffer. Samples were then further dialyzed into size exclusion chromatography buffer (10 mM Tris-HCl pH 7.3, 50 mM NaCl, 15 mM MgCl_2_, 1% glycerol) and resolved over a SuperDex 200/10/300 GL (GE Healthcare) using an ÄKTA FPLC (GE, 18-1900-26); desired fractions were pooled and stored at −80 °C.

### Assessment of contaminating proteolysis activity in SVP prep

For whole cell lysate, 1 mg of proteins from HeLa whole cell lysate was denatured in 8M Urea (Sigma-Aldrich), 50 mM Tris-HCl pH 7.0 for 10 minutes at 37 °C before being reduced in 1 mM Tris(2-carboxyethyl)phosphine (Life Technologies) for 10 minutes and then alkylated in 2 mM 2-chloroacetamide (Sigma-Aldrich) for 10 minutes in the dark. Samples were then diluted to a final concentration of 1M Urea, 50 mM NaCl (Sigma-Aldrich), 15 mM MgCl_2_ (Quality Biological) and 0.2M Tris-HCl pH 7.3. 5 μg of SVP was added to each sample and incubated for 2 hours at 37 °C. Samples were run on an in-house 6–10% SDS-PAGE gel and transferred to a nitrocellulose membrane. Total protein was visualized by ProAct membrane stain (Amresco) per the manufacturer’s instructions. For purified recombinant His-PARP1, 1 μg of His-PARP1 was automodified as previously published^5^ and then switched into the same buffer used for the whole cell lysate (described above) with or without 1M urea. 500 ng of SVP was used for each μg of PARP1 and digestion was carried out at 37 °C for 2 hours. Samples were run on in-house 6–10% SDS-PAGE gels and total protein was visualized by SimplyBlue Safe Stain (Life Technologies) per the manufacturer’s instructions. ^32^P-labeled PAR was visualized on a phosphor-screen (GE, BAS-III 2040) followed by imaging on a Typhoon FLA7000 (GE Healthcare Life Sciences).

### Expression and purification of Nudix hydrolases

The expression and purification have been previously published for the following hydrolases: *E. coli* NudF (*Ec*NudF/ADPRase/UniProtKB Q93K97)^18^*, E. coli* NudE (*Ec*NudE/ORF186/UniProtKB J7QI36)^32^, *Bdellovibrio bacteriovorus* (*Bd*3179/*Bd*NDPSase/UniProtKB Q6MIH8), *Deinococcus radiodurans Dr*1184 (*Dr*1184/ UniProtKB Q9RV46)^33^, *E.coli* RppH (*Ec*RppH/ORF176/UniProtKB P0A776^34^, *Agrobacterium tumefaciens* ORF147 (*At*ORF147/UniProtKB Q7CX66) and *Pseudomonas aeruginosa Pa3470 (Pa*3470/UniProtKB Q9HYD6)^35^. Homo sapiens NudT16 (*Hs*NudT16/UniProtKB Q96DE0) was expressed and purified as described in the methods from the Structural Genomics Consortium (http://www.thesgc.org/structures/3cou). For *Ec*RppH, the last step of purification yielded ≥90% homogeneity before being loaded onto a HiLoad 26/60 Superdex 200 prep grade gel filtration column (GE Healthcare), equilibrated in gel filtration buffer (50 mM Tris-HCl pH 8.5, 300 mM NaCl); fractions containing ≥95% homogeneity, as determined by SDS-PAGE, were pooled. *Ec*YfcD was purified using the method described for *Ec*ADPRase^18^.

### Structural analysis of Selected Nudix hydrolases

Selected Nudix nucleotide sugar hydrolases were used to test their activity against protein-conjugated ADPr: *E. coli* NudF, YfcD and NudE, and *Bdellovibrio bacteriovorus HD100* NDPSase. The structures were structurally aligned using SSM^36^ and rendered with PyMOL^37^. PAR was constructed and minimized in MOE software package (Chemical Computing Group, Montreal, Canada)^38^. For the sugar hydrolases, PAR was modeled in the active site taking into account the binding preference observed in the structures in complex with sugar nucleoside derivatives^18^.

Selected known single domain Nudix enzymes were structurally aligned using SSM^36^ and rendered using PyMOL^38^. PAR was modeled in the active site using a ‘template guide’, i.e. the mRNA present in the structure of the complex of *Ec*RppH (PDB ID 4S2X)^39^ and the IMP present in the structure of *Hs*NudT16 (PDB ID 2XSQ)^40^. Models of the complex structures were built using the selected structures and the PAR. Modeling steps were performed in MOE^38^. Hydrogen atoms and charges were added automatically to the protein. The binding site was defined as all protein atoms within 4.5 angstrom of any atom of bound ligand. A steepest descent energy minimization was used with a MMFF94x forcefield.

### Automodification of  WT and E988Q PARP1

Automodification was performed as published previously^5^ with the following changes: both WT and E988Q PARP1 were incubated with 0.6 μM (1.85 kBq/μL, 37 kBq/sample) ^32^P-NAD^+^ for 10 minutes at room temperature, following which WT PARP1 was incubated with 1 mM NAD^+^ (non-radioactive) for 10 minutes at room temperature to allow for polymer elongation.

### Hydrolysis of protein-conjugated ADPr to phosphoribose

For comparative analyses by SDS PAGE: 5 pmoles of 6xHis-PARP1 WT or E988Q mutant were exposed to a hydrolase (various enzymes and amounts) in hydrolysis buffer (50 mM Tris-HCl pH 7.0 (Thermo Scientific), 150 mM NaCl (Sigma-Aldrich), 15 mM MgCl_2_ (Quality Biological), 1 mM 3-aminobenzamide (Sigma Aldrich)) for two hours at 37 °C.

For comparative analyses by liquid chromatography-tandem mass spectrometry (LC-MS/MS): 60 pmoles of 6xHis-PARP1 was exposed to 120 pmoles of SVP or 3 nmoles of Nudix hydrolase (*Ec*RppH or *Hs*NudT16) in hydrolysis buffer for two hours at 37 °C.

### Protein digestions for LC-MS/MS analysis

Proteins were denatured in 8 M Urea, 50 mM Tris-HCl pH 7 for 10 minutes at 37 °C before being reduced in 1 mM Tris-(2-Carboxyethyl)phosphine for 10 minutes and then alkylated in 2 mM 2-chloroacetamide for 10 minutes in the dark. Samples were diluted to: 1M Urea, 50 mM NaCl, 15 mM MgCl_2_, 0.2 M Tris-HCl pH 7 (7.3 at room temperature), and 1 mM CaCl_2_. LysC (Wako) and Trypsin (Promega) were added at a 1: 50 enzyme:substrate ratio.

### Phosphoenrichment of phosphoribosylated peptides

The ion metal affinity chromatography (IMAC) method was performed as described previously^5^.

### LC-MS/MS analysis of phosphoribosylated peptides

Peptides were separated on a Thermo-Dionex RSLCNano UHPLC instrument with ~10 cm x 75 micron ID fused silica capillary columns with ~10 micron tip opening made in-house with a laser puller (Sutter) and packed with 3 micron reversed phase C18 beads (Reprosil-C18.aq, 120 Angstroms, Dr. Maisch). We applied a 90 min gradient of 10–35% B at 200 nL/min using 0.1% acetic acid as solvent A and solvent B of 0.1% acetic acid, 99.9% acetonitrile. MS data was collected with a Thermo Orbitrap Elite. Data-dependent analysis was applied using Top 5 selection and fragmentation was induced by CID and HCD. Profile mode data was collected in all scans. MS analysis parameters were as previously described^5^.

### Database search of MS/MS spectra for peptide and protein identification

Raw files were analyzed by MaxQuant version 1.5.3.8 using protein, peptide and site FDRs of 0.01 and a score minimum of 40 for modified peptides, 0 for unmodified peptides; delta score minimum of 17 for modified peptides, 0 for unmodified peptides. Sequences were searched against an in-house database containing the proteins of interest as well as UniProt *Escherichia coli* BL21 DE3 database (definitions updated October 15^th^, 2014). MaxQuant search parameters: Variable modifications included Oxidation (M), Acetylation (Protein N-term), carbamidomethyl (C), phosphorylation (STY) and phosphoribosylation (DEKRC). Max labeled amino acids was 3, max missed cleavages was 2, enzyme was Trypsin/P, and max charge was 7.

## Results

### Purification of Snake Venom Phosphodiesterase I from *Crotalus adamanteus* involves both affinity purification and size exclusion chromatography

**Figure 1:**
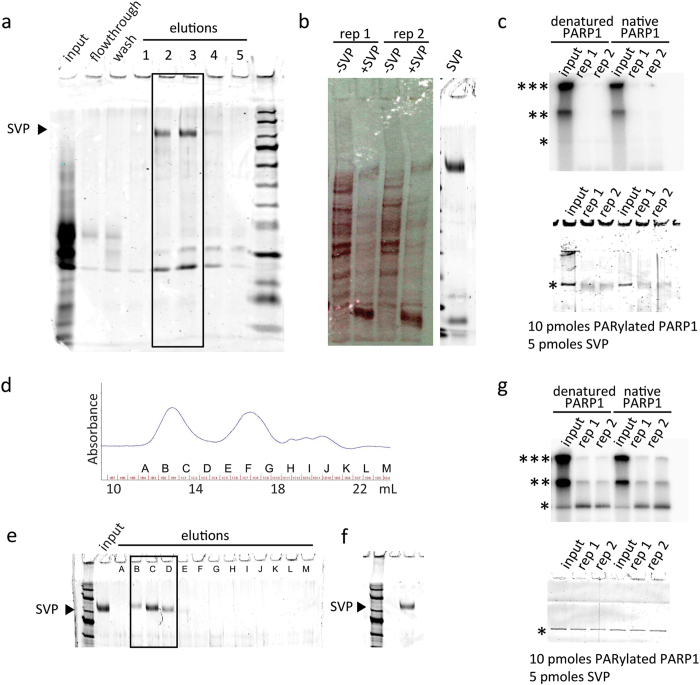
Purification of snake venom phosphodiesterase I for the digestion of protein-conjugated PAR. SVP was affinity purified on a blue sepharose column (boxed fractions in panel (**a**) producing a protease-contaminated product; (b and c), proteolysis is evident by the loss of protein bands following exposure to SVP). The addition of SVP results in protein degradation within lysates (**b**) and of purified PARP1 (**c**). The rightmost section in panel b indicates the size of SVP from the pooled fraction from (**a**). For (**b**) 1 mg of whole cell lysate was exposed to 50 pmoles of SVP for 2 hrs at 37 °C, pH 7. For (**c**) 10 pmoles of PARP1 was exposed to 5 pmoles of SVP for 2 hrs at 37 °C, pH 7. Size exclusion chromatography (**d,e**) results in a purified SVP fraction (**f**) that removed the contaminating protease activity (**g**). Top sections in panels c and g show the autoradiographs of PARylated PARP1 and the bottom sections show the corresponding coomassie staining of the gels. Reaction conditions for (**g**) were identical to those of (**c**). ***SDS-PAGE well, **SDS-PAGE interface between stacking and resolving gels, *native size of PARP1; rep = replicate.

Snake venom phosphodiesterase I (SVP) from *Crotalus adamanteus* was shown to degrade PAR nearly 50 years ago^41^ and has since proven a valuable tool for the degradation of PAR into its linear, branching and terminal subunits, a technique that yields quantitative information regarding the molecular structure of the intact polymer^42^,^43^. The utility of this enzyme, however, is greatly determined by the purification scheme employed to isolate it from the large number of proteases as well as phosphatases and nucleotidases present in the *C. adamanteus* venom^44^. Oka *et al.* successfully isolated the phosphodiesterase activity of commercially available SVP away from the contaminating phosphatase and 5′-nucleotidase activity through affinity purification using blue sepharose, a molecule which mimics NAD^+^ and therefore interacts with the active domain of SVP^45^. The results from a simplified version of this method used by our group is shown in Fig. 1a, where 150 mM potassium phosphate pH 7.5 is used as a single step elution off a blue sepharose column. This purification scheme paved the way for development of the quantitative method mentioned above to determine the structure of the intact polymer, but did not address the need to eliminate contaminating protease activity. This protease activity can be problematic when using SVP to hydrolyze protein-conjugated ADPr, either MAR or PAR, for the purpose of creating a phosphoribose ‘tag’ at the otherwise ADP-ribosylated amino acid residue^5^,^6^,^12^. Such protease activity is demonstrated in Fig. 1b wherein a complex mixture of proteins is exposed to blue sepharose purified SVP, resulting in the degradation of the target proteins and the appearance of SVP along with its co-purified proteins. This proteolytic activity is further shown against purified,^32^P-PARylated PARP1 (both native and denatured) in Fig. 1c. In order to separate the 115 kD SVP from the major contaminating proteins (<30 kD), we subjected the blue sepharose purified product to size exclusion chromatography, yielding a simple mixture of what we hypothesize to be the various glycolytic forms of SVP, based on the knowledge that most secreted proteins are glycosylated^46^, including those found in snake venom^47^ (Fig. 1d–f). When tested against ^32^P-PARylated PARP1 as in Fig. 1c, this highly pure form of SVP displayed phosphodiesterase activity without apparent proteolytic activity (Fig. 1g). Similar results were seen against whole cell lysate, allowing for use of this enzyme for ADP-ribosylation site identification by mass spectrometry^5^.

While the pipeline presented here is an effective method for isolating SVP from snake venom, we believe the complexity of the purification scheme, along with lot-to-lot variability observed from commercial sources of SVP which serve as the input for this purification (data not shown), stands to be greatly improved by the availability of a recombinant enzyme which could be reliably expressed, purified and scaled to meet the often large material demands of proteomic pipelines. For this reason, we went on to compare the activity of SVP with that of relatively small, stable and well-characterized Nudix hydrolases, which we hypothesized could hydrolyze protein-conjugated ADPr, either MAR or PAR, to phosphoribose with similar specificity.

### Nudix ADPrases do not hydrolyze protein-conjugated ADPr

**Figure 2:**
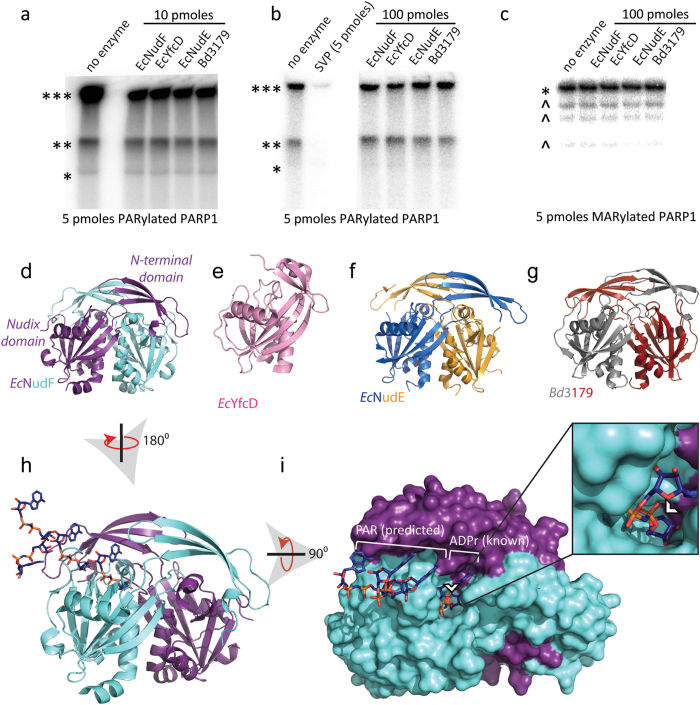
Nudix ADPrases are ineffective against protein-conjugated ADPr. (**a–c**) Autoradiograph showing ^32^P-labeled mono- or poly(ADP-ribose) conjugated to PARP1 following exposure to canonical nucleotide sugar hydrolases: *Ec*NudF, *Ec*YfcD and *Ec*NudE and *Bd*3179. For (**a**), 5 pmoles of PARylated PARP1 was exposed to 10 pmoles of hydrolase for 2 hrs at 37 °C; for (**b**), 100 pmoles of hydrolases were utilized, while in (**c**) 5 pmoles of PARP1 E988Q, a mutant only capable of synthesizing mono(ADP-ribose), was exposed to 100 pmoles of hydrolases for 2 hrs at 37 °C. Ribbon diagrams show the structure of each of the enzymes used: (**d**) *Ec*NudF/ADPrase (PDB ID 1KHZ)^18^, (**e**) *Ec*YfcD (PDB ID 2FKB), (**f**) *Ec*NudE (PDB ID 1VHG) and (**g**) *Bd*3179 (PDB ID 5C7Q)^52^. Panel (**h**) shows a surface representation of *Ec*NudF with modeled PAR polymer based on the complex with a nonhydrolyzable ADPr^48^. Panel (**i**) shows a surface representation of *Ec*NudF with the modeled PAR polymer, the inset shows how the terminal ribose is protected and buried within the enzyme. The white arrowhead indicates where protein conjugation would occur on ADPr. ***SDS-PAGE well, **SDS-PAGE interface between stacking and resolving gels, *native size of PARP1, ^co-purified PARP1 protein fragments.

Nudix ADPrases are responsible for the breakdown of free ADPr into its phosphoribose and adenosine monophosphate subunits, thus modulating the levels of free ADPr^18^,^48^. This knowledge lead us to test a group of Nudix ADPrases for hydrolase activity against protein-conjugated MAR and PAR: PARP1, an enzyme known to autoPARylate in the presence of NAD^+^, was exposed to ^32^P-labeled NAD^+^ producing either ^32^P-labeled PARylated (on WT PARP1) or MARylated (on the catalytically deficient PARP1 E988Q mutant) proteins to serve as substrates for hydrolysis by candidate Nudix enzymes or the positive control, SVP (Fig. 2a–c). As Fig. 2b shows, the various Nudix enzymes tested did not significantly hydrolyze ADPr from its conjugated proteins when compared to the positive control SVP. From a structural perspective, the lack of activity towards ADPr could be explained by the dimeric structure of ADPrases, where each dimer is formed by monomers of an N-terminal β-sheet domain and a C-terminal Nudix domain (Fig. 2d–g). The N-terminal domains are swapped, creating two active sites where both monomers contribute to substrate recognition (each active site is composed of the N-terminal β-sheet of one monomer and the C-terminal Nudix domain of the other monomer). As shown in Fig. 2h,i, ADPr is nested in the active site of the ADPRase *Ec*NudF so that the 1′-hydroxyl of the terminal ribose group is completely buried by the protein dimer (white arrowhead in Fig. 2i), preventing conjugation to another ADPr group (or a protein). This explanation could likely be extended to the other three nucleoside sugar hydrolases tested in this study as they display the same quaternary arrangement and have a high structural homology with a pairwise root mean square deviation ranging from 0.9 to 2.0 Å (Fig. 2d–g; rmsd calculated with SSM^36^).

### Single domain Nudix hydrolases are capable of hydrolyzing protein-conjugated ADPr

**Figure 3:**
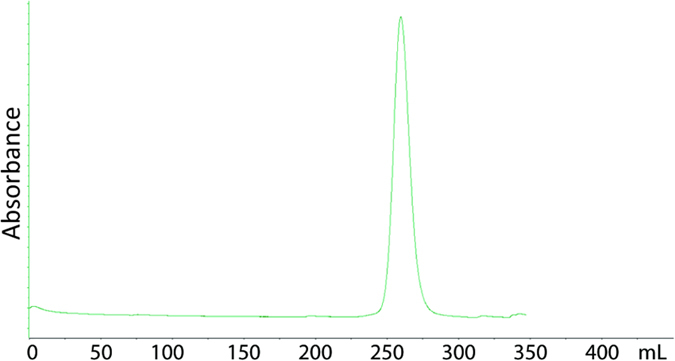
Sizing chromatography of the Nudix enzyme *Ec*RppH. *Ec*RppH size exclusion chromatography uses 50 mM Tris-HCl pH 7.5, 1 mM EDTA, 0.30 M NaCl at a flow rate of 2.0 mL/min; peak at 259.4 mL on a HiPrep 26/60 Superdex 200 prep grade. Molecular weight standards thyroglobulin Mr 670,000, peak 140.6 mL; γ-globulin Mr 158,000, peak 184.5 mL; ovalbumin Mr 44, 000, peak 226.2 mL; myoglobin Mr 17,000, peak 253.4 mL; vitamin B12 Mr 1350, peak 297.3.

**Figure 4:**
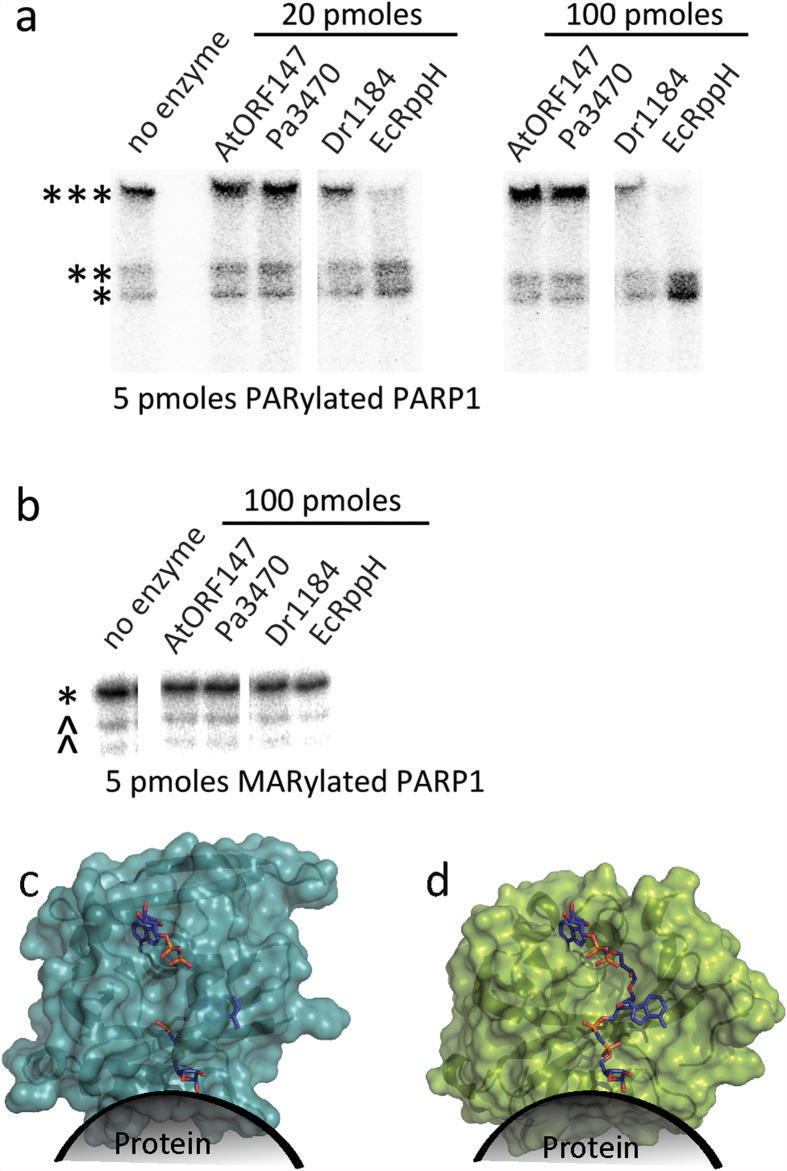
Single domain Nudix hydrolases *Ec*RppH and *Dr*1184 show activity against protein-conjugated ADPr. (**a,b**) Autoradiograph showing the ^32^P-labeled PAR (**a**) or MAR (**b**) conjugated to PARP1 following exposure to Nudix hydrolases: *At*ORF147, *Pa*3470, *Dr*1184, and *Ec*RppH. For (**a**), 5 pmoles of PARylated PARP1 was exposed to either 20 or 100 pmoles of hydrolase for 2 hrs at 37 °C. For (**b**), 5 pmoles of MARylated PARP1 E988Q mutant was exposed to 100 pmoles of hydrolase for 2 hrs at 37 °C. (**c,d**) show surface representations of *Dr*1184/CoAse (teal, PDB ID 1NQY) (**c**) and *Ec*RppH (green, PDB ID 4S2Y) (**d**) with PAR modeled into their active sites based on the orientation of capped mRNA bound to *Ec*RppH in the reported structure^39^. A dark shadow shows the possible orientation of the attached PARylated protein. ***SDS-PAGE well, **SDS-PAGE interface between stacking and resolving gels, *native size of PARP1, ^co-purified PARP1 protein fragments.

In order to consider Nudix enzymes with active sites more open to fit the target ADPr group bound to either a PAR polymer or protein, we turned to Nudix enzymes representative of families which are not swapped dimers and lack N- or C-terminal domain insertions, hypothesizing that enzymes with just the Nudix fold would have a more open active site. We chose four Nudix enzymes known to be monomeric by gel filtration^35^ (e.g. RppH as shown in Fig. 3). Two of them (*Dr*1184/CoAse from *Deinococcus radiodurans* and *Ec*RppH from *Escherichia coli*) degraded the ^32^P-labeled PAR on the model protein PARP1 (Fig. 4a), while only *Ec*RppH showed slight activity against ^32^P-labeled MAR at the tested concentration (Fig. 4b). Structural analysis and modeling revealed that the active site within these enzymes could accommodate protein-conjugated ADPr (Figs. 4c,d), as opposed to the dimeric ADPrases (c.f. Fig. 2d–g).

### Both *Ec*RppH and *Hs*NudT16 can degrade protein-conjugated PAR and MAR to a phosphoribose tag for mass spectrometry

**Figure 5:**
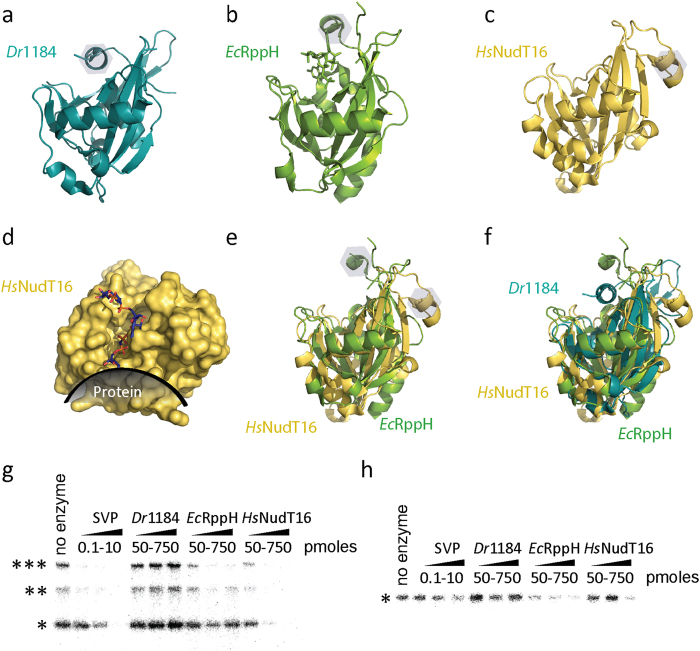
*Hs*NudT16 degrades protein-conjugated ADPr. (**a**) A ribbon model of the structure of *Dr*1184/CoAse (teal, PDB ID 1NQY). (**b**) A ribbon model of the structure of *Ec*RppH (green, PDB ID 4S2Y). (**c**) A ribbon model of *Hs*NudT16 (PDB ID 2XSQ), (**d**) Surface representation of *Hs*NudT16 with PAR modeled (sticks) and a protein depicted at the conjugation site of ADPr. Panel (**e**) shows a structural alignment between *Hs*NudT16 and *Ec*RppH, while (**f**) shows a structural alignment of *Hs*NudT16, *Ec*RppH and *Dr*1184. The grey hexagons in panels (**a**), (**b**), (**c**) and (**e**) show the helix that moves upon substrate binding. Panels (**g**) and (**h**) show the removal of ^32^P-labeled ADPr from 5 pmoles of PARylated (**g**) or MARylated (**h**) PARP1 by increasing amounts of SVP (0.1, 1 and 10 pmoles) or *Dr*1184, *Ec*RppH or *Hs*NudT16 (50, 250 and 750 pmoles). ***SDS-PAGE well, **SDS-PAGE interface between stacking and resolving gels, *native size of PARP1.

A recent study by Palazzo *et al.*^13^ has revealed that *Hs*NudT16, a human Nudix (deoxy)inosine diphosphatase^49^ which is also known to decap small nucleolar RNAs^50^ as well as cytoplasmic mRNAs^51^, has the ability to degrade protein-conjugated ADPr. As shown in Fig. 5a–f *Hs*NudT16 has a high structural similarity to both *Ec*RppH and *Dr*1184 (which showed activity against protein-conjugated ADPr, see Fig. 4a,b) and also possesses an open active site which would allow for the target ADPr to be conjugated to a protein or additional ADPr unit(s). Based on these similarities, we postulated that the activity against protein-conjugated ADPr would be comparable for all three Nudix enzymes. To test this,^32^P-PARylated or MARylated PARP1 was exposed to increasing amounts of SVP, *Ec*RppH, *Dr*1184 or *Hs*NudT16. As shown via autoradiographs in Fig. 5g,h, both *Hs*NudT16 and *Ec*RppH are able to hydrolyze both protein-conjugated PAR and MAR.

**Figure 6:**
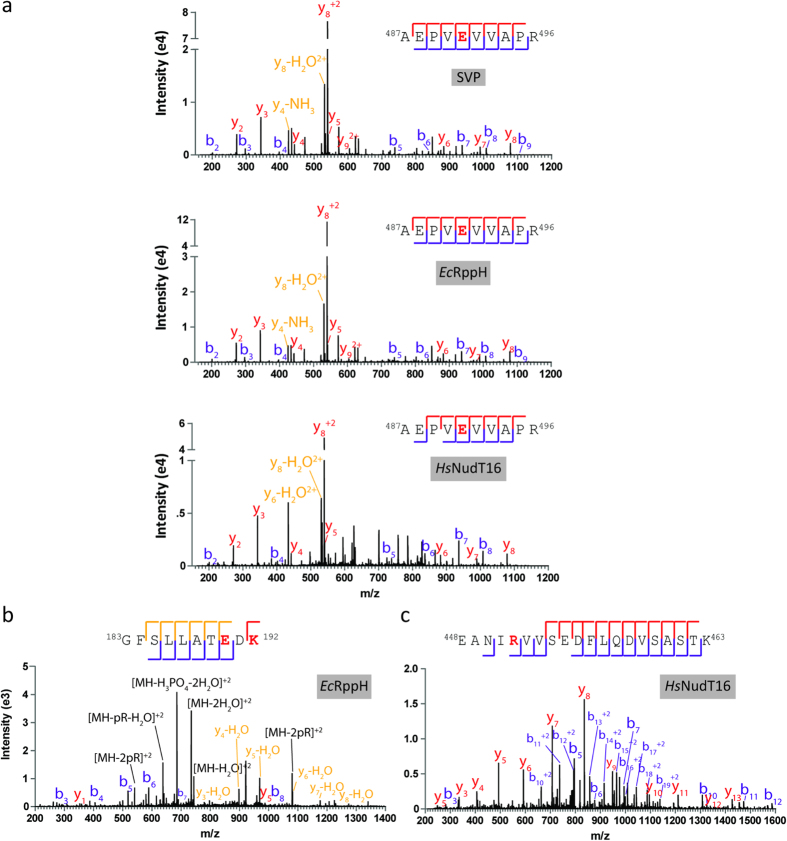
Treatment of PARylated PARP1 protein with SVP, *Ec*RppH and *Hs*NudT16 results in the identification of phosphoribosylation sites by mass spectrometry. (**a**) E491 (red, identified as 212 Dalton mass shift) on PARP1 was identified as a PARP1 automodification site following SVP, *Ec*RppH and *Hs*NudT16 treatment. (**b**) E190 and K192 were identified as PARP1 automodification sites on PARP1 following *Ec*RppH digestion. (**c**) Identification of R452 as a PARP1 automodification site following *Hs*NudT16 treatment.

To validate that *Ec*RppH and *Hs*NudT16 are degrading protein-conjugated ADPr down to its phosphoribose attachment site, we treated 60 pmoles of automodified WT PARP1 with 120 pmoles of SVP, 3 nmoles of *Ec*RppH or 3 nmoles of *Hs*NudT16 (either 2x or 50x molar excess over PARP1, as per the activity assays shown in Fig. 5) before digesting the proteins to peptides and subjecting them to phosphoenrichment on an IMAC matrix^5^. As shown in Table 1, phosphoribosylation sites were confidently identified by mass spectrometry on PARP1 following treatment by each enzyme, demonstrating that all three enzymes are hydrolyzing the ADPr modification down to its phosphoribose attachment site. Notable differences were observed between the site identifications that were achieved following the use of these three enzymes — only two of the 14 phosphoribosylated peptide species were found in all samples, and all enzymes produced at least one unique peptide species. Taken together, these data suggest that these enzymes have an inherent bias for PTM structure, location, or length and therefore may be used in parallel to achieve greater coverage of the ADP-ribosylated proteome. Representative spectra from these runs have been annotated in Fig. 6, further confirming the evidence of phosphoribose modifications on these peptides.

## Discussion

**Figure 7:**
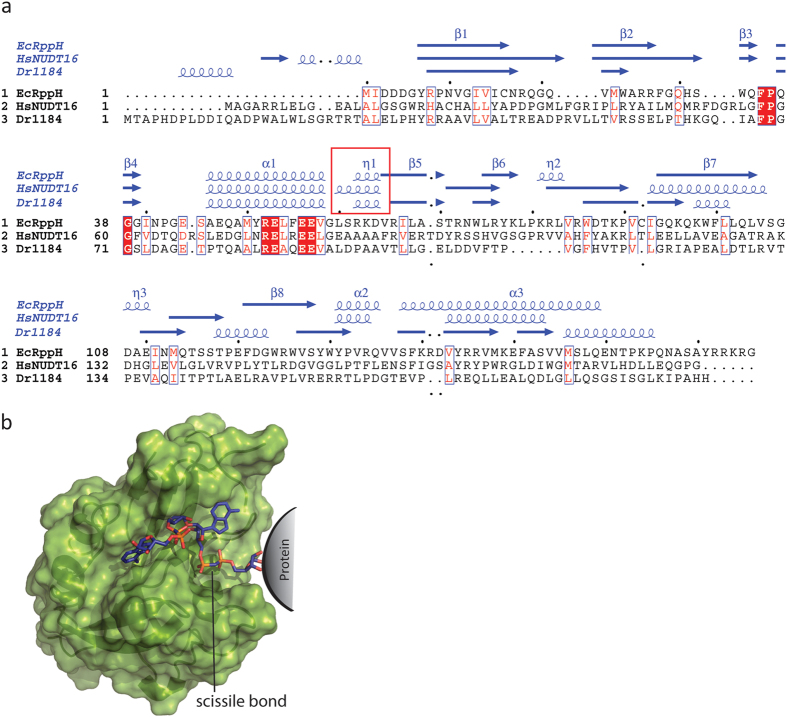
Alignment of Nudix enzymes with activity against protein-conjugated PAR reveals a novel 3_10_ helix. (**a**) Alignment of the sequences of *Ec*RppH, *Hs*NudT16 and *Dr*1184. The red background indicates sequence identity, red letters sequence indicates conservation of charge, and white background indicates non-homologous residues. The secondary structure of each enzyme is shown in blue. The box indicates a short 3_10_ helix not observed in other Nudix enzymes such as the ADPRases. The secondary structure elements are more conserved between the two enzymes with activity towards PAR and MAR (*Ec*RppH and *Hs*NudT16) compared to *Dr*1184 that can only hydrolyze PAR. (**b**) Surface representation of the model of *Ec*RppH with PAR modeled and minimized using the software package MOE. The diphosphate bond of the terminal ADP-ribose attached to a protein is in the active site, well positioned to be hydrolyzed and released as a phosphoribosylated protein product, where the scissile bond is marked.

This work, along with that of Palazzo *et al.*^13^, has revealed new substrates for the RNA decapping Nudix hydrolases *Ec*RppH and *Hs*NudT16, as well as the CoAse *Dr*1184, though it must be noted that the existence of this activity against protein-conjugated ADPr *in vivo* has yet to be explored. In an attempt to determine the structural basis for this unique protein-conjugated ADPr hydrolase activity, we aligned these three proteins with respect to their known structures and identified a short, novel 3_10_ helix downsteam from the helix of the Nudix signature sequence (Fig. 7a, red box), suggesting a possible significance to these structures for the degradation of protein-conjugated ADPr. We caution, however, that the structure is not known of the other two single domain Nudix hydrolases tested which lack the activity of interest: *At*ORF147 and *Pa*3470 (c.f. Fig. 4a,b). Moreover, the sequence homology between *Ec*RppH/*Hs*NudT16/*Dr*1184 and *At*ORF147/*Pa*3470 is not high beyond the Nudix signature sequence, so it is difficult to predict if the presence or absence of such short helices are the determining structural factor to facilitate the hydrolysis of protein-conjugated ADPr. Modeling of a trimeric PAR in the binding site of *Ec*RppH positions the ribose towards the front of the enzyme allowing ample space for a protein to bind (Fig. 7b). Notably, the bond to be cleaved to leave a phosphoribose attached to a protein is in an ideal position to be hydrolyzed by the residues of the catalytic site of the Nudix enzyme (Fig. 7b). This model gives compelling structural arguments to the biochemistry data collected.

Mass spectrometry-based proteomics represents the gold standard for the study of post-translational modifications, and the field of ADP-ribosylation will surely benefit from increased access to the suite of proteomic tools that have been developed for other PTMs, such as phosphorylation, acetylation and ubiquitylation. The advent of tag-based approaches for identifying ADP-ribosylation sites has begun to provide access to these tools, but adoption has been relatively low due to technical difficulties which accompany the current methods. The work presented here promises to streamline one of the major methods for ADPr site identification: the simplification of PAR or MAR to its phosphoribose attachment. Recombinant *Hs*NudT16 and *Ec*RppH can be synthesized and purified from *E. coli*, allowing for low-cost, high yield production which can be performed in most proteomic laboratories. Furthermore, since the structure is known for both of these enzymes, it is possible to predict mutations and truncations which could potentially increase the enzyme’s activity towards protein-conjugated ADPr; for example, introducing mutations which will further open up the active site to allow larger ADP-ribosylated substrates access.

The work presented here has the potential to greatly aid in the elucidation of the ADP-ribosylated proteome by providing researchers the necessary tools for generating phosphoribose as a mass spectrometry friendly site localization tag of protein ADP-ribosylation sites.

## Additional Information

**How to cite this article**: Daniels, C. M. *et al.* Nudix hydrolases degrade protein-conjugated ADP-ribose. *Sci. Rep.* **5**, 18271; doi: 10.1038/srep18271 (2015).

## Figures and Tables

**Table 1:**
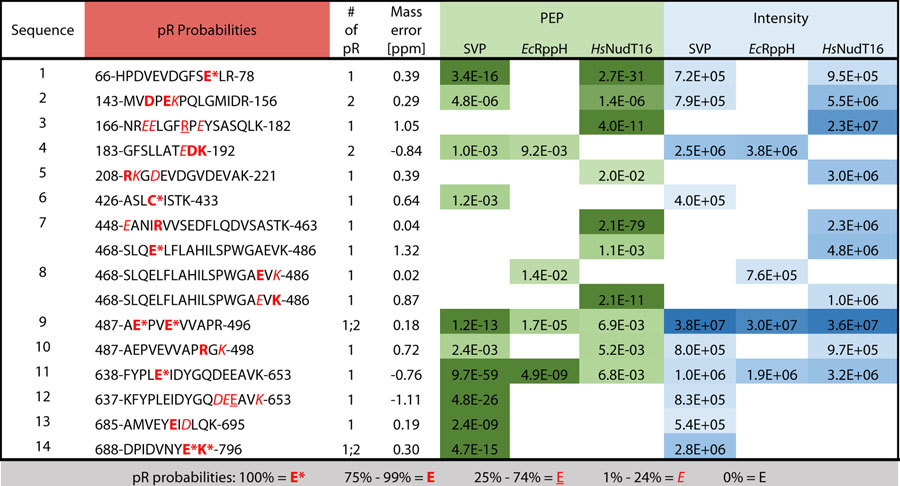
Phosphoribosylated PARP1 peptides identified by LC-MS/MS following hydrolysis of PARylated PARP1 by SVP, *Ec*RppH and *Hs*NudT16.

Phosphoribosylated peptides reported with their corresponding posterior error probabilities (PEP) and summed intensities from 60 pmoles of the model protein PARylated PARP1 after the conversion of ADPr to phosphoribose by 120 pmoles of SVP, 3 nmoles of *Ec*RppH or 3 nmoles of *Hs*NudT16. All elements of the table are found in the pR (DEKRC)Sites.txt output table from MaxQuant. Summed intensities include all forms of the peptide (for example, both the singly and doubly modified forms of peptides 9 and 14), PEPs are the most confident reported for each peptide, and mass errors [ppm] are the largest mass errors reported for each peptide identification included in this table.
